# Clinical evaluation of iron treatment efficiency among non-anemic but iron-deficient female blood donors: a randomized controlled trial

**DOI:** 10.1186/1741-7015-10-8

**Published:** 2012-01-24

**Authors:** Sophie Waldvogel, Baptiste Pedrazzini, Paul Vaucher, Raphael Bize, Jacques Cornuz, Jean-Daniel Tissot, Bernard Favrat

**Affiliations:** 1 Blood Transfusion Service of the Swiss Red Cross, Lausanne, Switzerland; 2 Department of Ambulatory Care and Community Medicine, University Hospital of Lausanne, Lausanne, Switzerland; 3 Department of Community Medicine, Ambulatory Care, and Emergencies, University of Geneva, Switzerland

## Abstract

**Background:**

Iron deficiency without anemia is related to adverse symptoms that can be relieved by supplementation. Since a blood donation can induce such an iron deficiency, we investigated the clinical impact of iron treatment after a blood donation.

**Methods:**

One week after donation, we randomly assigned 154 female donors with iron deficiency without anemia, aged below 50 years, to a four-week oral treatment of ferrous sulfate versus a placebo. The main outcome was the change in the level of fatigue before and after the intervention. Aerobic capacity, mood disorder, quality of life, compliance and adverse events were also evaluated. Hemoglobin and ferritin were used as biological markers.

**Results:**

The effect of the treatment from baseline to four weeks of iron treatment was an increase in hemoglobin and ferritin levels to 5.2 g/L (*P *< 0.01) and 14.8 ng/mL (*P *< 0.01), respectively. No significant clinical effect was observed for fatigue (-0.15 points, 95% confidence interval -0.9 points to 0.6 points, *P *= 0.697) or for other outcomes. Compliance and interruption for side effects was similar in both groups. Additionally, blood donation did not induce overt symptoms of fatigue in spite of the significant biological changes it produces.

**Conclusions:**

These data are valuable as they enable us to conclude that donors with iron deficiency without anemia after a blood donation would not clinically benefit from iron supplementation.

**Trial Registration:**

ClinicalTrials.gov: NCT00981877

## Background

Oral iron treatment in non-anemic iron-deficient subjects can have beneficial effects on fatigue and physical performance. The first evidence was provided 50 years ago [1]. Further studies using fatigue questionnaires and serum ferritin as a marker have confirmed this effect [2-4]. Physiological measurements have also been carried out in randomized double-blind controlled trials: aerobic capacity increases [5-8] and muscle fatigability decreases [9] among trained or untrained volunteers.

Iron deficiency without anemia (IDWA) is not a contraindication for blood donation, although highly prevalent among menstruating women. Studies show that 22% of women of childbearing age have a ferritin level of less than 15 ng/mL and 4% have iron deficiency anemia [10]; and between 6% and 27% of female blood donors eligible for donation (that is, non-anemic) have iron deficiency, depending on donation frequency [11]. A whole blood donation of 450 mL contains around 55 g to 70 g of hemoglobin and consequently 187 mg to 238 mg of iron. This amount is between one and two thirds of the ideal store for a woman, who could give blood three times a year without any substitution, according to European Council recommendations [12]. However, normal diet does not compensate quickly enough for iron loss through blood donations [13] and even a 16-week iron-rich diet encouraged by professional counselors has only a moderate effect on IDWA [14].

Some authors advocate iron replacement after donation to prevent iron depletion, especially as donors could be symptomatic [15-17]. According to an observational survey, fatigue is the most common systemic adverse symptom which follows blood donation, affecting 11% of female and 4% of male blood donors [18]. Recent prospective studies have proven that iron supplementation versus a placebo allows donors to donate more frequently, but did not consider the clinical benefit for the donor [19-21]. Moreover, the design of these studies could not distinguish between IDWA and iron deficiency anemia after donation because, at the initiation of iron replacement, only pre-donation values of hemoglobin and ferritin were available. However, the treatment of IDWA can have an impact on well-being or work efficiency, as suggested in a non-randomized controlled study [22].

The present study aimed to determine, in a randomized controlled trial, the effect of iron treatment on fatigue after blood donation among menstruating female blood donors presenting with IDWA.

## Methods

### Design

This trial was a four-week, double-blind, placebo-controlled, parallel group, randomized trial with a 1:1 allocation ratio.

Physicians working at the Blood Transfusion Service were responsible for seeing all potential participants and controlling eligibility criteria. Once informed consent forms were signed, a blood donation was performed. Approximately 450 mL of venous blood was collected within a blood pack set, allowing pre-donation sampling from which around 4 mL were used for our study.

### Setting

Donors coming for a whole blood donation at the Lausanne Blood Transfusion Centre of the Swiss Red Cross were recruited. Randomization and follow-up took place at the Department of Ambulatory Care and Community Medicine of Lausanne University Hospital.

### Eligibility

Female donors aged 18 to 50 years and eligible for a blood donation according to national regulations were asked to participate. Exclusion criteria were psychiatric conditions or diseases that rendered the participant unable to give consent; thyroid, hepatic, rheumatic, kidney, cardiopulmonary, or intestinal disease; acute or chronic inflammation; diabetes; hemochromatosis; pregnancy; medical treatment that could alter iron absorption and any iron supplementation.

### Intervention

Volunteers self-administered either 80 mg/day oral ferrous sulfate (FeSO_4_; Tardyferon, Robapharm, Boulogne, France) or placebo for four weeks. To decrease side effects, the pills could be taken during a meal; Verdon *et al. *showed a significant decrease in fatigue without drop-out for side effects using the same recommendation [4]. Iron pills were given in an electronic drug monitoring system (Medication Event Monitoring System (MEMS), Aardex Europe, Switzerland [23]). The iron treatment and placebo were identical in appearance and taste.

### Randomization, allocation, and concealment

Randomization took place a week after the blood donation with the following criteria for inclusion: hemoglobin level ≥ 120 g/L, ferritin level ≤ 30 ng/mL. A simple random allocation sequence without restriction was generated by an independent pharmacy according to a pre-established computer-generated list. Each drug package was identified with a unique number according to the randomization schedule and given to the nurse in charge of the participant. The code was held by the pharmacist and remained unbroken until the end of the trial. The allocation remained concealed from participants, care providers, investigators and the statistician until the end of the statistical analysis.

### Outcomes

The primary outcome was the level of fatigue perceived by donors, scored at baseline (randomization) and after four weeks on a 10-point visual analogue scale (VAS) ranging from 'no fatigue' (0) to 'very severe fatigue' (10). A self-administered validated questionnaire evaluating subjective fatigue with a Likert scale was also used - the Fatigue Severity Scale (FSS) [24]. The score was obtained by averaging responses from nine questions each ranging from 1 to 7, increasing with the severity of fatigue. Both fatigue scores were also measured just before donation.

Additional clinical outcomes were measured at baseline and after treatment. Any change in aerobic capacity was measured using a step test (Chester step test), which has demonstrated excellent repeatability and a good correlation with maximal oxygen uptake (r = 0.92) [25]. Depression and quality of life were assessed using the Prime-MD [26] and the SF-12 [27] self-questionnaires, respectively.

A blood count was measured from venous samples exclusively, using an auto-analyzer (Sysmex XE 2100; Sysmex Corporation, Kobe, Japan) and ferritin concentration was determined by an immunoturbidimetric assay (Tina-quant; Roche Diagnostics, Mannheim, Germany), from the first milliliter of the donation, one week after donation, and again after the intervention. To exclude elevated ferritin levels caused by acute phase, C-reactive protein was also measured at randomization. We stated empirically that a value higher than 20 mg/L would be considered as a significant inflammation, that is, an exclusion criterion.

To explore bias related to menorrhagia, the pictorial chart of Janssen *et al. *was used at randomization [28].

An electronic system (MEMS) recorded the date and time each time the vial was opened. Subjects were asked to use this electronic pill-bottle for each dose and to swallow the dose immediately after opening. A study that used this system has shown that compliance and motivation to take the treatment were thus improved [29]. Questions were asked at the end of the intervention to evaluate whether the electronic device was properly used. Unused pills were also counted. Medication adherence was calculated as the number of days with at least one opening of the electronic device divided by the total number of monitored days. Finally, participants were asked to guess to which group they had been assigned.

### Statistical analysis

The main outcome variable was the level of fatigue at four weeks. The sample size for randomized volunteers was calculated using a two-sample comparison of means to detect a one-point difference between the groups on the VAS, similar to the minimal clinically appreciable difference for pain [30]. According to a previous study, a standard deviation of two points was to be expected [4]. For a two-tailed test (α = 0.05, power = 0.80) and anticipating a 10% dropout rate, we calculated a total sample size rounded to 140 participants.

Analyses were by intention-to-treat. The null hypothesis was that there was no difference in fatigue VAS scores between the experimental and control groups at four weeks, adjusted for the baseline level of fatigue on the same scale. Effects of treatment were measured using linear regression, with fatigue levels at four weeks as the dependent variables and group allocation and fatigue at baseline as independent variables. The measures of effect for the secondary outcomes were assessed by the same method. A significant level of treatment effect was set at *P *< 0.05, using a likelihood ratio test. Missing data from dropouts were not replaced and only donors who were followed-up after four weeks' treatment were included in the analysis. All calculations were performed with StataCorp 2008, Statistical Software: Release 10.0, Stata Corporation, College Station, Texas, USA.

### Ethical considerations

The study was approved in July 2008 by the University of Lausanne Ethics Committee for Clinical Research (131/08) and the Swiss Agency for Therapeutic Products (2008DR4328). Subjects presenting with anemia one week after the donation were not randomized and received 80 mg/day FeSO_4 _over three months. The published protocol remains valid since no amendment was necessary [31].

## Results

### Population characteristics

**Figure 1:**
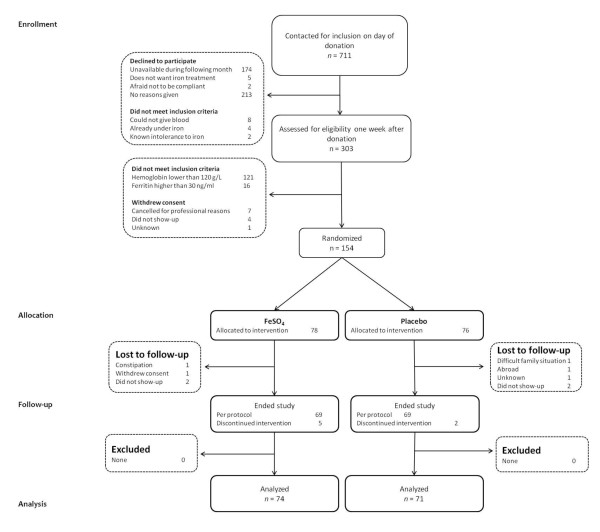
**Flow chart**.

**Figure 2:**
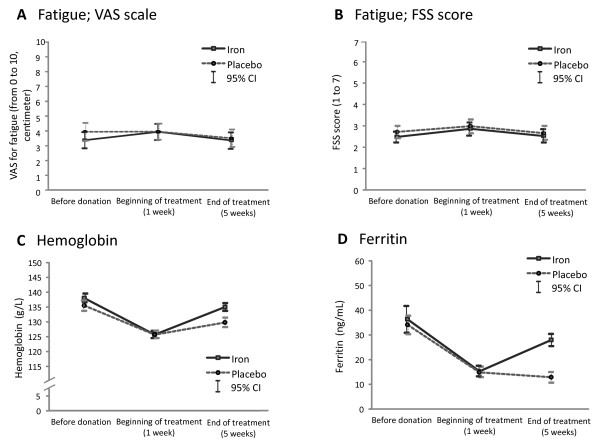
**Variations over time in fatigue, hemoglobin and ferritin among randomized volunteers**.

Between November 2008 and September 2010, 711 female donors were invited to participate. Of these, 154 donors presenting with IDWA were allocated to either placebo or iron: 17 first-time donors and 137 consecutive donors whose mean (range) number of donations was 8 (0 to 55). Reasons for non-eligibility, refusals and dropouts are provided in Figure 1. Randomization ensured that the groups were similar at baseline for all measures except for the pictorial bleeding test, where women from the intervention group tended to have less menorrhagia than those from the control group (Table 1). Nine (12.2%) donors from the treatment group and four (5.6%) from the placebo group reported amenorrhea. Figure 2 reports variations over time for both groups before and after allocation. One week after the donation, we observed a significant decrease in hemoglobin (mean = -12.9 g/L, standard deviation (SD) = 6.5 g/L, *P *< 0.0001) and serum ferritin (mean = -19.2 ng/mL, SD = 0.86, *P *< 0.0001) concentrations. A mean change in fatigue one week after donation and before allocation was significant but lower than one point on the FSS scale (mean = 0.27, SD = 1.2, *P *= 0.0001), but not significant on the VAS scale (mean = 0.17, SD = 2.6, *P *= 0.257).

**Table 1:** Baselines characteristics.

	FeSO_4_^a^ n = 74	Placebo n = 71	Difference^a^ Absolute values
**Age**, mean years (SD)	32.9 (8.4)	30.7 (8.8)	2.1
**Number of previous donations per year**, n (%)			
None	26 (35.1%)	25 (35.2%)	-0.1%
One	29 (39.2%)	31 (43.7%)	-4.5%
Two	19 (25.7%)	15 (21.1%)	4.6%
**Weight**, mean kg (SD)	64.2 (10.7)	67.6 (13.3)	-3.4
**Pictorial Bleeding Assessment chart**			
Score > 185, n (%)	6 (8.2%)	9 (12.7%)	-4.5%^a^
**Before donation**; mean (SD)			
Visual analogue scale for fatigue	3.4 (2.4)	3.9 (2.6)	-0.6
Fatigue severity scale	2.5 (1.1)	2.7 (1.2)	-0.2
Hemoglobin, g/L^b^	138 (6.3)	135 (7.5)	3
Ferritin, ng/mL^b^	36.3 (22.4)	34.1 (15.0)	2.2
**One week after donation**; mean (SD)			
Visual analogue scale for fatigue	3.9 (2.3)	4.0 (2.4)	-0.02
Fatigue severity scale	2.9 (1.3)	3.0 (1.4)	-0.1
Vitality score (SF-12V2)^c^	53.1 (12.9)	55.9 (11.3)	-2.8
Chester step test, mLO_2_/kg/min	37.0 (7.2)	36.9 (5.9)	0.1
Hemoglobin, g/L	126 (5.2)	126 (5.3)	-0.02
Ferritin, ng/mL	15.3 (7.7)	14.8 (7.3)	0.4
C-reactive protein, mg/L	2.1 (2.9)	2.9 (3.5)	0.8
Depression (PHQ-9), n (%)	4 (5.4%)	4 (5.7%)	-0.3%
Mental health (SF-12_NL_)^d^	38.6 (4.4)	39.3 (5.3)	-0.7
Physical condition (SF-12_NL_)^d^	53.7 (4.2)	53.6 (4.2)	0.09

^a^Clinically significant difference. P-values of differences were not calculated, since randomization and allocation ensured that any difference could only be due to chance. ^b^Hemoglobin and ferritin values before donation were not available for two donors from the control group. ^c^One donor from the intervention group and two from the control group did not answer the 10^th ^question on the SF-12V2. ^d^SF-12 was incomplete for three donors in each group. SD: standard deviation.

### Effects on outcomes

Iron supplementation had a significant effect on biological markers but not on fatigue or aerobic capacity (Table 2). Complete outcome data were not available for nine randomized donors; therefore full application of intention to treat was not possible. Nevertheless, results were confirmed by worst and best case scenario analyses. An absence of effect on the clinical outcome was confirmed in a per-protocol analysis including 69 participants in each group (confidence interval 95%, -0.71 to 0.74; *P *= 0.967). Effects on the physical condition score of the SF-12 were mainly due to less interference of pain with normal work (*P *= 0.003), and less limitations in work or other activities as a result of physical health (*P *= 0.012) being reported in the FeSO_4 _group. The effect of treatment on depression was inconclusive given the low number of donors with depression at four weeks (one in the FeSO_4 _group versus two in the placebo group).

**Table 2:** Outcomes in iron and placebo groups after four weeks of treatment.

	FeSO_4_	Placebo	Treatment effect^a^		
			
	n = 74 mean (SD)	n = 71 mean (SD)	Crude ITT group difference Δ (95%CI)	Significance level *LR test*	Adjusted effect^b^ Δ (CI95%)
					
Visual analogue scale fatigue	3.4 (2.4)	3.5 (2.5)	-0.15 (-0.9 to 0.6)	*P *= 0.697	-0.18 (-0.9 to 0.6)
Fatigue severity scale^c^	2.5 (1.3)	2.6 (1.5)	-0.06 (-0.4 to 0.3)	*P *= 0.760	-0.05 (-0.4 to 0.3)
Vitality item (SF-12V2)^d^	53.6 (12.7)	55.3 (12.3)	-0.24 (-3.9 to 3.4)	*P *= 0.897	-0.13 (-3.8 to 3.6)
					
Chester step test, mLO_2_/kg/min^c^	40.5 (14.5)	40.1 (17.0)	0.28 (-4.5 to 5.1)	*P *= 0.907	0.02 (-4.8 to 4.8)
					
Hemoglobin, g/L	135 (6.7)	130 (5.3)	5.2 (3.5 to 6.9)	*P *< 0.001	5.3 (3.7 to 7.0)
Ferritin, ng/mL	28.0 (9.8)	12.9 (8.3)	14.8 (12.2 to 17.4)	*P *< 0.001	15.1 (12.6 to 17.6)
Quality of life (SF-12_NL_)^e^					
Physical condition	54.8 (3.3)	52.4 (5.2)	2.4 (1.1 to 3.7)	*P *< 0.001	2.4 (1.1 to 3.7)
Mental health	40.1 (4.8)	40.7 (4.8)	-0.4 (-2.0 to 1.2)	*P *= 0.590	-0.5 (-2.0 to 1.1)

^a^Treatment effect was measured using linear regression, with treatment group and baseline value of fatigue as independent variables. ^b^Was adjusted for baseline imbalance for menstrual bleeding. ^c^Data were missing for one donor from the control group. ^d^Two donors from the intervention group and four donors from the control did not answer question 10 from the SF-12V2. ^e^SF-12 was not completed by nine donors from the intervention group and three from the control. CI: confidence interval; ITT: intention to treat analysis; LR: likelihood ratio.

The proportion of donors whose hemoglobin concentration returned to that recorded before blood donation was similar in both groups (28.4% in FeSO_4 _versus 25.3% in placebo; *P *= 0.711). On the other hand, 13 (18.3%) donors from the placebo group, and two (2.7%) from the treatment group (*P *= 0.002) had lower hemoglobin concentrations four weeks after treatment than one week after donation, three of which from the placebo group became anemic (*P *= 0.115). Furthermore, after four weeks of treatment, 2.7% of donors under FeSO_4 _had blood concentrations of ferritin below 12 ng/m L compared to 57.7% in the placebo group (*P *< 0.001). Mean aerobic capacity increased both in the treatment group (from 37.0 to 40.5 mLO_2_/kg/min; *P *= 0.0002), and in the placebo group (from 36.9 to 40.1 mLO_2_/kg/min; *P *= 0.014).

### Adverse events and adherence to treatment

No serious adverse event was reported. Undesirable events mentioned included gastrointestinal symptoms (n = 33), dizziness (n = 3), headache (n = 2), acne (n = 2), palpitations (n = 1), and renal lithiasis (n = 1). The differences between treatment and placebo are reported in Table 3. Medication adherence was 96% and similar in both groups. Seven participants interrupted their treatment prematurely, two of which, one in each group, did so because of a side effect.

**Table 3:** Undesirable events, compliance, and blinding.

	FeSO_4_ n = 74	Placebo n = 71	Absolute difference	Significance level *Fisher's exact test*
**Undesirable events**, n (%)				
Hard stools	13 (17.6%)	3 (4.2%)	13.4%	*P *= 0.015
Liquid stools	9 (12.2%)	3 (4.2%)	8.0%	*P *= 0.130
Abdominal pain	7 (9.5%)	4 (5.6%)	3.9%	*P *= 0.534
Nausea	2 (2.7%)	0 (0%)	2.7%	*P *= 0.497
Any gastrointestinal	25 (33.8%)	8 (11.3%)	22.5%	*P *= 0.001
Other events	6 (8.1%)	3 (4.2%)	3.9%	*P *= 0.495
Any event	29 (39.2%)	11 (15.5%)	23.7%	*P *= 0.002
**Days with correct dosing **(compliance)^a^				
Mean (SD)	26.3 (3.9)	26.5 (2.8)	-0.2	*P *= 0.624
Median (range)	27 (7 to 35)	27 (13 to 35)	0	
**Believed to have received**^b ^n (%)				
FeSO_4_	44 (60.3%)	9 (12.9%)	37.4%	*P *< 0.001
Placebo	13 (18.8%)	42 (60.0%)	41.2%	
Does not know	16 (21.9%)	19 (27.1%)	5.2%	

^a^The container with remaining pills was not returned by two donors from the control group. ^b^One patient from the intervention group did not answer.

## Discussion

In this randomized double-blind controlled trial, a four-week iron treatment of IDWA initiated one week after a blood donation had no beneficial effect on fatigue and consistently did not improve aerobic capacity, despite having a significant impact on hemoglobin and ferritin levels. Furthermore, a blood donation does not induce significant fatigue measured one week after donation. This study was sufficiently powered to exclude a clinically significant effect of iron supplementation on fatigue. Consequently, these data provide important information on the well-being of donors: blind iron-supplementation after donation is not justified even if it has been shown that adverse events related to a blood donation penalize blood supply [32]. Taking these data into consideration, we have decided not to introduce iron replacement for young female donors at our transfusion center, since no clinical benefit has been documented. However, further trials focusing on long-term iron deficiency or chronic fatigue among donors could lead to a change in our policy.

Most participants of this study were made iron deficient by a single blood donation while all previous experimental studies included participants with long-term IDWA induced by a progressive imbalance between intake and loss of iron [2-9]. Indeed, the median pre-donation ferritin level of donors randomized in our study (34 ng/mL) was above the threshold of an overt iron deficiency (12 ng/mL to 15 ng/mL) [33] and IDWA was induced by acute bleeding. Interestingly, our results suggest a difference in clinical responses to short-term and long-term IDWA. Such a rapid transition to IDWA possibly has no effect on non-erythroid compartments, such as nervous tissue or muscle. In this context, our results do not conflict with data from the recent non-controlled trial that showed numerous clinical benefits of iron treatment after donation, reducing fatigue, prostration, difficulty in concentrating, headache, hair loss and nail breakage [22]. Besides the methodological limits of this study, donors treated with iron already had IDWA before donation since their inclusion criterion was a pre-donation level of ferritin of < 10 ng/mL. These donors were therefore more likely to have iron deficits in non-erythroid compartments before blood donation.

However, comparing only biological changes between groups, significantly more donors in the placebo group had a decreased ferritin (*P *< 0.001) and hemoglobin (*P *= 0.002) level during intervention. Consequently, we should not neglect that iron treatment could prevent symptomatic deterioration of iron status related to further donations.

The total quantity of elemental iron (2,200 mg) orally administered to each participant in our study was set according to iron loss from a donation. While this is certainly not sufficient to compensate for all occurrences of IDWA, the main purpose of this study was to investigate the clinical effect of iron deficiency induced by a single blood donation. Overall mean changes of hemoglobin (Δ 11 g/L) and ferritin (Δ 13 ng/mL) levels between baseline and the end of the treatment were consistent with expected values. Such a biological change, induced by a comparable amount of elemental iron, was enough in some previous randomized placebo controlled trials to obtain a favorable impact on fatigue and endurance [4-6,8,9]. The degree of hypoferritinemia could also be critical. Indeed Verdon *et al. *showed that the treatment effect on fatigue depended on baseline ferritin levels and was not quantitatively significant among subjects with a ferritin level above 50 ng/mL [4]. However, even if the cut-off level of ferritin used for inclusion in our study was not severe (< 30 ng/mL), the mean ferritin value before treatment was 15 ng/mL, which is comparable to other randomized placebo controlled trials dealing with IDWA and showing a clinical improvement after iron treatment [3,5,6,9].

Blood donors come spontaneously to the donation center and are then clinically selected by professionals as being adequately healthy and fit to donate. Consequently, symptoms of fatigue should not be very frequent, as notably observed with half of the donors reporting a level of fatigue before donation of three or less on the VAS. Moreover, blood donation did not induce clinically significant fatigue [34] in our study and the minor difference detected reflected rather a regression toward the mean due to a natural fluctuation of fatigue. Therefore, treatment effect on fatigue was possibly absent merely because no symptom was perceived before intervention. Indeed, our randomized controlled trial had the particularity to exclusively use biological inclusion criteria to evaluate treatment effect on fatigue among subjects with IDWA. Furthermore, a measurement of aerobic capacity, which is probably more appropriate for healthy volunteers, did not show any significant treatment effect either, thus strengthening our result on fatigue.

Our study suggests that oral FeSO_4 _administered to donors with IDWA improves quality of life. Surprisingly, this isolated effect is exclusively related to physical condition. More precisely, the main item of significance concerned pain. To our knowledge there are no studies reporting a significant effect of iron treatment on pain and no physiological basis can support the link between iron treatment and pain. Consequently, despite its significance, this result has been considered as spurious.

Our study showed significant side effects in the treatment group. This significance resulted mainly from hardening of stools (absolute difference: 13%, *P *< 0.01), which could be considered rather as a slight discomfort. Moreover, despite this significant side effect, drop-out rate for a side effect, adherence to treatment and correct guessing of treatment group were similar for each group. Interestingly, Bruner *et al. *showed no difference in side effects, particularly concerning constipation, between treatment and placebo, in spite of a higher daily dose of FeSO_4 _(260 mg of elemental iron daily) and a proportion of subjects in the iron treatment group that correctly guessed their group assignment (62%) similar to that of our trial [35]. According to other clinical trials lacking in a placebo, constipation related to oral FeSO_4 _is the most frequent adverse effect, ranging from 11% in a study comparing intravenous versus oral iron among postpartum patients [36] to 30% of new cases in a study testing an older population (mean age 62 years) [37]. Among donors, this side effect seems to occur less frequently: 3% to 13% [22,38]. Our significant result on these moderate side effects adds, however, an argument against broad-based supplementation after each donation.

Our study had several limitations. Firstly, outcomes of this study were restricted to fatigue, physical performance, mood disorder and quality of life but did not include other consequences of IDWA that could affect the well-being of donors. Treatment of IDWA has been shown to improve cognitive function in randomized controlled trials [35,39]. Moreover, in a recently reported prospective clinical trial among blood donors, restless legs syndrome was frequent (18%) and iron treatment after donation was effective [38]. Concerning hair loss, evidence that iron treatment is beneficial is still lacking [40], but data from Pittori *et al. *suggest the beneficial impact of oral iron treatment [22]. Secondly, our study revealed that anemia was a highly prevalent form of iron deficiency (44%) after donation, which contrasts with data obtained from a comparable cohort of menstruating women in the general population [10]. Indeed, 121 donors were excluded from randomization because of anemia and received a three-month iron treatment. No follow-up data are available since the aim of our study was exclusively to explore IDWA. Anemia one week after a blood donation is not surprising since our national recommendation for the hemoglobin threshold for such a donation is 120 g/L. Female participants who became anemic one week after donation were also observed in a study by Rosvik *et al*., even in the iron-treatment group (one week of oral iron, 100 mg/day) and in spite of an older mean age (43.2 years; SD = 12.1) and a higher pre-donation hemoglobin level (137 g/L; SD = 0.7) [21]. According to Fowler's data, around 75% of donors return to their initial hemoglobin level after eight weeks but the other subjects need a longer recovery period of up to 15 weeks [41]. Anemia induced by a blood donation may be causative of disabling symptoms and it would be fair to explore this clinically. Thirdly, we cannot exclude that clinical effect of treatment was not detected because follow-up took place too early. Indeed, Pittori *et al. *observed a significant decrease in fatigue after six months of follow-up, but not after only two months [22].

## Conclusions

This randomized controlled trial has shown no clinical benefit of treating IDWA induced by a single blood donation. Moreover, significant fatigue induced by a blood donation has not been observed. This first clinical information concerning iron deficiency among donors is reassuring but strongly prompts further clinical trials, extended to iron deficiency anemia after donation, to ensure progress in the management of blood donors.

## Competing interests

BF gave lectures to both Pierre Fabre Médicament and Vifor Pharma, companies that might have an interest in the submitted work. The other authors have no competing interest.

## Authors' contributions

SW, BP, PV, RB and BF designed the study. Statistical analysis was carried out by PV. SW, PV and BF interpreted the results. SW drafted the manuscript. BP, PV, RB, JDT, JC and BF revised the manuscript and approved the final version.

## Pre-publication history

The pre-publication history for this paper can be accessed here:

http://www.biomedcentral.com/1741-7015/10/8/prepub
